# Genotypic Characterisation of Carbapenem-Resistant Enterobacteriaceae in a Tertiary Care Hospital in South India

**DOI:** 10.7759/cureus.75032

**Published:** 2024-12-03

**Authors:** Sowmya Anbazhagan, Arvindh Krishnan E, Divya S, Mathavi Sureshkumar

**Affiliations:** 1 Microbiology, Vinayaka Mission's Kirupananda Variyar Medical College and Hospital, Salem, IND; 2 Medical Microbiology, Vinayaka Mission's Kirupananda Variyar Medical College and Hospital, Salem, IND

**Keywords:** antimicrobial resistance, carbapenem-resistant, enterobacteria, genotypic, south india population

## Abstract

Introduction

The antimicrobial resistance of *Enterobacteriaceae* is variable and is influenced by both geographic location and regional antibiotic use. The overuse of antibiotics, especially in hospitalised patients, suppresses the growth and persistence of drug-resistant bacteria. This study aimed to detect the prevalence of carbapenem-resistant *Enterobacteriaceae* and the genes responsible for the resistance.

Methods

A cross-sectional study has been conducted over the course of two years, from October 2021 to September 2023. A total of 2,152 samples, including pus, blood, urine, sputum and various body fluids, were collected and subjected to study. All data were analysed and presented as frequency with percentage.

Results

Out of 2,152 samples, 659 (32.1%) samples showed growth. Among them, 250 (38%) were found to be *Enterobacteriaceae*, of which 22 (8.8%) were resistant to carbapenems. The isolates were *Escherichia coli* (nine, 40%) followed by *Klebsiella pneumoniae* (eight, 36%), *Morganella morganii* (two, 9%), *Klebsiella oxytoca* (one, 5%), *Proteus vulgaris *(1, 5%), and *Enterobacter cloacae* (1, 5%). NDM (14, 63.63%) was the most common gene detected from the isolates.

Conclusion

Our research leads us to the conclusion that resistance to carbapenem medication can result from either the generation of carbapenemase or from non-carbapenemase mechanisms like loss of porin channels or an increase in the efflux pump. According to our research, the primary source of carbapenem resistance is metallo-β-lactamase. Therefore, it is critical for all the laboratories to identify the mechanism and incidence of carbapenem resistance in order to support epidemiological research, infection control and antibiotic stewardship.

## Introduction

The widespread use of carbapenems in healthcare settings has led to the emergence of carbapenem-resistant *Enterobacteriaceae* (CRE) as a significant issue in high-risk patients [1]. The antimicrobial resistance of *Enterobacteriaceae* is variable and is influenced by both geographic location and regional antibiotic use. The primary cause of decreased antibiotic absorption is altered porin expression linked to the overexpression of β-lactamases with low carbapenemase activity and the acquisition of carbapenemase genes that code for the enzymes that break down carbapenems. The other two are efflux pump mechanisms and decreased permeability of the outer membrane to the medication [2]. Carbapenemases were thought to be species-specific, chromosomally encoded β-lactamases until the early 1990s. Once the genes for carbapenemases were found on transposons and plasmids, it became apparent that these enzymes may spread horizontally. Since then, a wide range of carbapenemases have been characterised, and the strains that produce the enzyme have been reported and spread over the world [3].

Bacterial enzymes known as β-lactamases hydrolytically render inactive β-lactam antibiotics, including penicillins, monobactams, carbapenems and cephalosporins, which act as a primary factor contributing to the rise of resistant bacteria [4]. The four classes A, B, C and D of β-lactamases are based on sequence homology. β-lactamases are classified into classes A, C and D, and these are the enzymes with a serine base that create a covalent acyl-enzyme intermediate. The mechanism by which class B β-lactamase, a metallo-β-lactamase (MBL), breaks and activates the β-lactam ring is dependent on a water molecule linked to a divalent cation (Zn2+).

Class A carbapenemases most likely originated from ambient microbiota that carried genes encoding for enzymes that hydrolyse carbapenems [5]. Enzymes from KPC, SFC, NMC-A and GES, IMI and SME are among them. β-lactamase inhibitors, such as clavulanic acid, exhibit little activity against class C and D SBLs and no activity against the majority of serine or metallo-carbapenemases, although they are active against numerous class A serine β-lactamases [6]. Additionally, they are inert against MBLs; more recently, non-β-lactam drugs, such as avibactam, have an extended spectrum of activity against classes A, C and D SBLs, including KPC-2 [6].

There are nearly 22 KPC variants. Among these variants, KPC-2 and KPC-3 are more commonly reported from clinical infections. KPC-3 leads to a slight increase in hydrolysis of ceftazidime than the other KPC-1 and KPC-2 variants [7]. Class B (MBL) has a broad spectrum of hydrolytic activity, including all β-lactam antibiotics except monobactam. They include NDM, VIM, IMP and SPM-1 families and require zinc or any other heavy metal for catalysis. These are not inhibited by mechanism-based inhibitors like tazobactam, clavulanate and sulbactam, but they are inactivated by metal chelators such as EDTA [8]. Class C carbapenemases, which include CMY-2, ACT-1, CMY-10 and DHA-1, confer resistance to penicillins and cephamycins such as cefotetan and cefoxitin [9]. β-lactam and β-lactamase inhibitor combinations and cephalosporins are not inhibited by clinically used drugs like β-lactamase inhibitors such as clavulanic acid [10].

Of the approximately 455 enzymes in class D β-lactamases (OXA), only a few variations are regarded as carbapenemases. They were found in clinical isolates of *Acinetobacter*; they weakly hydrolyse carbapenems and are not well inhibited by clavulanate. Compounds that are not generated from β-lactams, such as avibactam, have a wider range of β-lactamases, including classes A, C and D [11]. This study aimed to determine the prevalence of CRE and identify the genetic mechanisms underlying their resistance.

## Materials and methods

Study design and setting

This was a two-year cross-sectional study conducted between October 2021 and September 2023 at the Department of Microbiology, Vinayaka Mission’s Kirupananda Variyar Medical College and Hospitals, Salem, Tamil Nadu, India. The study aimed to determine the prevalence of CRE and to identify the genetic mechanisms responsible for resistance. Ethical clearance for the study was obtained from the Institutional Ethical Committee, with the approval reference number VMKVMCH/IEC/21/045. The hospital, serving as a tertiary care centre, provided a diverse range of clinical samples from both inpatient and outpatient departments, ensuring a comprehensive representation of CRE prevalence in the region.

Inclusion criteria

The study included all isolates of Enterobacteriaceae that demonstrated reduced susceptibility to carbapenems, specifically with an imipenem and meropenem zone size of ≤19 mm, as determined by the Clinical and Laboratory Standards Institute (CLSI) guidelines. These isolates were collected from various clinical specimens, including urine, blood, sputum, pus, wound swabs, and body fluids such as pleural and ascitic fluid.

Exclusion criteria

Isolates that were non-Enterobacteriaceae, as well as Enterobacteriaceae sensitive to carbapenems (imipenem and meropenem), were excluded from the study. This exclusion ensured that the analysis focused solely on CRE to maintain the specificity and relevance of the study's objectives. Additionally, environmental isolates or repeat samples from the same patient were excluded to avoid duplication and ensure accurate epidemiological representation.

Study procedure 

Various samples like pus, urine, wound swab, ascitic fluid, pleural fluid, endotracheal aspirate, throat swab, vaginal swab, blood and sputum were collected from the patients’ (in and out) departments under strict sterile aseptic precautions. All the samples were labelled properly with the name of the patient, type of specimen, and collection time and immediately transported or within two hours to the laboratory. Gram staining was done directly from the samples, and the samples were inoculated onto MacConkey agar and blood agar plates and incubated aerobically at 37°C overnight. The tests, which included Gram staining, catalase test, oxidase test, and motility testing by hanging drop, were done for preliminary identification of the organism. After preliminary identification of the organisms, speciation was done with biochemical reactions like the indole test, citrate utilisation test, urease test, mannitol motility medium, and triple sugar iron test. Antimicrobial susceptibility testing (AST) was done on the Mueller-Hinton agar plate by the Kirby-Bauer disc diffusion method and incubated aerobically for 16-18 hours at 37°C. Interpretations were made according to CLSI guidelines. Zone diameters of ≤19 mm for meropenem and imipenem were taken, and phenotypic confirmation was done by VITEK-2 (bioMérieux, Marcy-l'Étoile, France), which is a widely used commercial antimicrobial susceptibility test system. VITEK-2 is an integrated system that automatically performs rapid identification using algorithms based on fluorescence and colourimetry and AST based on kinetic analysis of growth data. 

Genotypic characterisation

Genotyping of CRE was performed using real-time polymerase chain reaction (RT-PCR), which is considered the gold standard method. Among the 22 carbapenem-resistant isolates, the genes blaKPC, blaVIM, blaNDM or blaOXA were detected under RT-PCR. RT-PCR technique is considerably simple and fast with respect to the standard PCR technique and is used for the rapid detection and identification of a variety of infectious and non-infectious pathogens and genes. PCR is used to amplify a targeted DNA sequence by use of hydrolysis probes that are short oligonucleotides that have a fluorescent reporter dye attached to the 5' end and a quencher dye to the 3' end. DNA was extracted by using the Hi-PCR® Carbapenemase Gene (Multiplex) Probe PCR Kit (HiMedia Laboratories Pvt Ltd, Thane, India). The recommended PCR program is as follows: initial denaturation at 95°C for 10 minutes followed by denaturation at 95°C for five seconds (no. of cycles: 45). Then annealing and extension done at 60°C for one minute and final holding temperature at 4°C. A cycle threshold (Ct) value of ≤40 was considered positive. From carbapenem-resistant isolates, the genes detected were NDM, KPC, VIM, OXA-51, OXA-23 and OXA-58. The device used was QuantStudio 3 RT-PCR system (Thermo Fisher Scientific, Waltham, MA) with Master Mix (HiMedia Laboratories Pvt Ltd, Thane, India) - HiMedia’s Hi-PCR® Carbapenemase Gene (Multiplex) Probe PCR Kit (MBPCR132). The protocol used for the PCR mix is given in Table 1.

**Table 1:** Protocol for PCR mix used in the study The components listed were manufactured by HiMedia Laboratories Pvt Ltd, Thane, India. PCR, polymerase chain reaction

Components	Volume to be added	
	CRG1 tube (μL)	CRG2 tube (μL)
Hi-Quanti 2X Realtime PCR Master Mix	12.5	12.5
CRG1 Primer-Probe Mix	4	-
CRG2 Primer-Probe Mix	-	4
Internal Control Primer-Probe Mix	1	1
Internal Control B DNA	1	1
Molecular Biology Grade Water for PCR	1.5	1.5
Positive Control/Negative Control/Template DNA	5	5
Total Volume	25	25

A Ct value of ≤40 for all four genes (NDM, KPC, IMP and VIM) alongside an internal control (IC) value of ≤40 is interpreted as positive for the presence of these genes. A Ct value of ≤40 for all four genes (OXA-51, OXA-23, OXA-48 and OXA-58) combined with an IC value of ≤40 confirms a positive result for the presence of these genes. In contrast, Ct values > 41 for all four genes and an IC value of ≤ 40 indicate a negative result. Similarly, if there are no Ct values for any of the genes or the IC, the result is deemed a failure due to the absence of amplification.

Statistical analysis 

Data were entered in Excel (Microsoft® Corp., Redmond, WA) and analysed with SPSS 26.0 (IBM SPSS Statistics for Windows, IBM Corp., Armonk, NY). Frequencies and percentages were calculated for categorical variables (e.g., prevalence of carbapenem resistance, organism distribution, gene detection). Mean and standard deviation (SD) were reported for continuous variables (e.g., sample counts by source).

## Results

In our study, a total of 250 *Enterobacteriaceae* were found, and the majority of isolates were found to be from urine 110 (44%), followed by pus (83, 33.2%), blood (27, 10.8%), sputum (17, 6.8%) and endotracheal aspirate (13, 5.2%). By the Kirby-Bauer disk diffusion method, 24 isolates were resistant to carbapenem, while VITEK-2 confirmed 22 isolates as carbapenem-resistant. CRE was more prevalent in male gender (12, 59%) than female gender (nine, 41%). Males have risk factors of urethral strictures, prostate enlargement, renal and ureteric stones and urinary incontinence, which leads to continuous exposure to a health care facility, high antibiotic usage and ICU admission, which can result in the acquisition of CRE infection. From 45-65 years of age, the organisms were predominantly isolated from pus (11, 50%) followed by urine (seven, 32%), blood (two, 9%) and endotracheal aspirate (two, 9%). Among 22 CRE isolates, the majority of organisms were *Escherichia coli *(nine, (40%), followed by* Klebsiella pneumoniae* (eight, 36%), *Morganella morganii* (two, 9%), *Klebsiella oxytoca* (one, 5%), Proteus vulgaris (one, 5%), and *Enterobacter cloaca*e (one, 5%). The genes detected were 14 NDM, three KPC, nine VIM, four OXA-51, 13 OXA-23 and three OXA-58. Seven isolates were exclusive MBL producers; five isolates were exclusively class D carbapenemase producers, and 10 isolates were co-producers of MBL and classes A and D carbapenemases.

**Figure 1:**
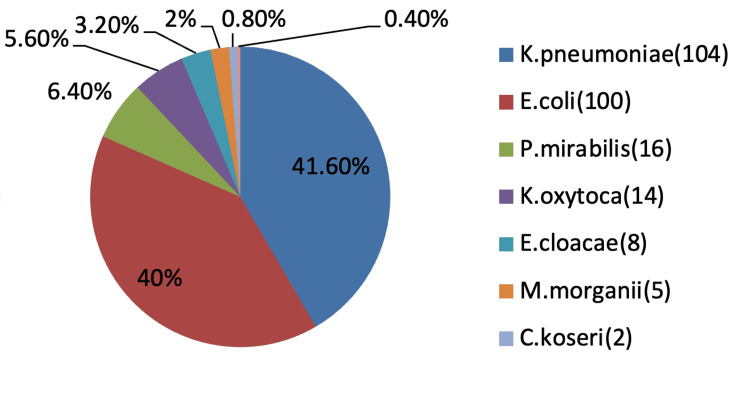
Distribution of Enterobacteriaceae in relation to organisms isolated (n = 250)

Among 250 isolates, the predominant organisms were *Klebsiella pneumoniae *(104, 41.6%), followed by *Escherichia coli *(100, 40%), *Proteus mirabilis* (16, 6.4%), *Klebsiella oxytoca* (14, 5.6%), *Enterobacter cloacae* (eight, 3.2%), *Morganella morganii *(five, 2%), *Citrobacter koseri* (two, 0.8%) and *Citrobacter freundii* (one, 0.4%), as shown in Figure 1.

Table 2 summarises the distribution of CRE among the total isolates of *Enterobacteriaceae* (n = 250) obtained in the study. The most prevalent CRE species identified was *Escherichia coli* (nine isolates, 40.90%), followed closely by *Klebsiella pneumoniae* (eight isolates, 36.36%). Less commonly identified organisms included *Morganella morganii *(two isolates, 9.09%), *Klebsiella oxytoca* (one isolate, 4.54%), *Proteus mirabilis* (one isolate, 4.54%) and *Enterobacter cloacae* (one isolate, 4.54%). No carbapenem resistance was observed in isolates of *Citrobacter koseri* or *Citrobacter freundii*.

**Table 2:** Distribution of carbapenem-resistant Enterobacteriaceae (CRE) among the total isolates of Enterobacteriaceae (n = 250)

Organisms	Total Isolates (n = 250)	No. of Isolates (n = 22)	%
Escherichia coli	100	9	40.90
Klebsiella pneumoniae	104	8	36.36
Morganella morganii	5	2	9.09
Klebsiella oxytoca	14	1	4.54
Proteus mirabilis	16	1	4.54
Enterobacter cloacae	8	1	4.54
Citrobacter koseri	2	-	-
Citrobacter freundii	1	-	-

Among 22 carbapenem-resistant isolates, the genes detected were 14 (63.6%) NDM, three (13.6%) KPC, nine (40.9%) VIM, four (18.2%) OXA-51, 13 (59.1%) OXA-23, and three (13.6%) OXA-58. Seven (31.8%) isolates were exclusive MBL producers, five (22.7%) isolates were exclusively class D carbapenemase producers and 10 isolates were co-producers of MBL and classes A and D carbapenemases. Table 3 illustrates the distribution of carbapenemase genes among various CRE isolates. The NDM gene was the most frequently detected, with *Escherichia coli* accounting for eight isolates (57.14%) and *Klebsiella pneumoniae* contributing five isolates (35.71%), followed by *Morganella morganii *with one isolate (7.14%). The OXA-23 gene was predominantly observed in *Escherichia coli* with six isolates (46.15%) and *Klebsiella pneumoniae* with five isolates (38.46%), while *Morganella morganii* and *Proteus mirabilis* each had one isolate (7.69%). The VIM gene was equally distributed between *Escherichia coli *and *Klebsiella pneumoniae*, each contributing four isolates (44.44%), with one isolate (11.11%) found in *Proteus mirabilis*. For the OXA-51 gene, there was equal representation with one isolate (25.00%) each in *Escherichia coli*, *Klebsiella pneumoniae*, *Proteus mirabilis *and *Klebsiella oxytoca*. The KPC gene was detected in *Klebsiella pneumoniae*, *Morganella morganii*, and *Enterobacter cloacae*, each with one isolate (33.33%). Similarly, the OXA-58 gene was identified in *Klebsiella pneumoniae*, *Morganella morganii *and *Klebsiella oxytoca*, each contributing one isolate (33.33%). This distribution emphasises the predominance of NDM and OXA-23 genes, especially in *Escherichia coli* and *Klebsiella pneumoniae*, while showcasing the varied presence of other carbapenemase genes across multiple species.

**Table 3:** Organism-wise distribution of carbapenemase genes (n = 22)

Gene	Escherichia coli	Klebsiella pneumoniae	Morganella morganii	Proteus mirabilis	Klebsiella oxytoca	Enterobacter cloacae	Total
NDM	8 (57.14%)	5 (35.71%)	1 (7.14%)	0 (0.00%)	0 (0.00%)	0 (0.00%)	14 (100%)
OXA-23	6 (46.15%)	5 (38.46%)	1 (7.69%)	1 (7.69%)	0 (0.00%)	0 (0.00%)	13 (100%)
VIM	4 (44.44%)	4 (44.44%)	0 (0.00%)	1 (11.11%)	0 (0.00%)	0 (0.00%)	9 (100%)
OXA-51	1 (25.00%)	1 (25.00%)	0 (0.00%)	1 (25.00%)	1 (25.00%)	0 (0.00%)	4 (100%)
KPC	0 (0.00%)	1 (33.33%)	1 (33.33%)	0 (0.00%)	0 (0.00%)	1 (33.33%)	3 (100%)
OXA-58	0 (0.00%)	1 (33.33%)	1 (33.33%)	0 (0.00%)	1 (33.33%)	0 (0.00%)	3 (100%)

**Figure 2:**
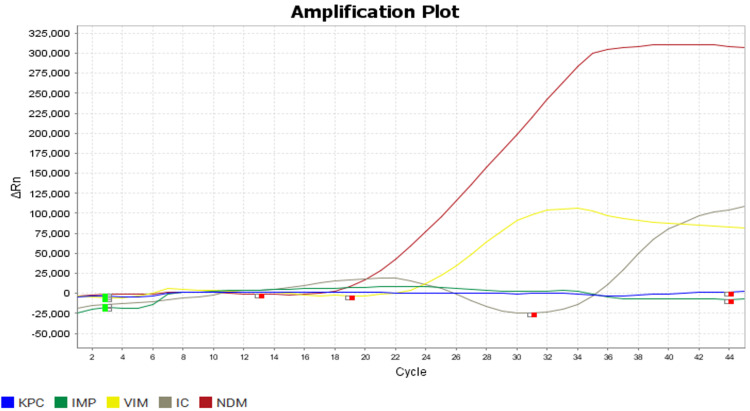
Polymerase chain reaction (PCR) graph showing the detection of NDM gene

**Figure 3:**
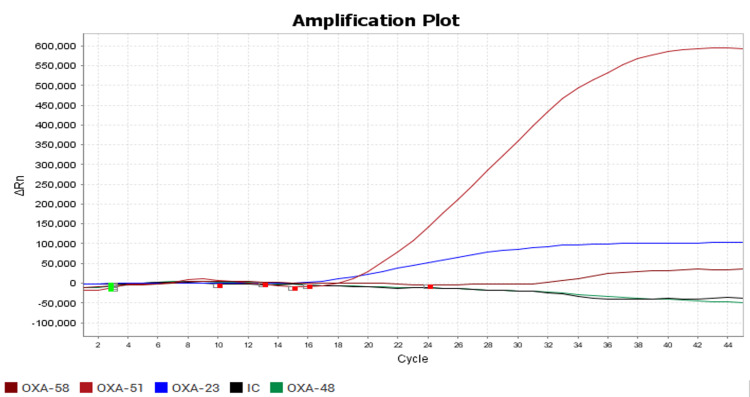
Polymerase chain reaction (PCR) graph showing the detection of OXA gene

In Figure 2, the amplification curve for the NDM gene shows a consistent increase in fluorescence intensity, crossing the Ct value of ≤40, confirming the presence of the NDM gene in the tested isolates. Similarly, Figure 3 illustrates the amplification curve for the OXA-51 gene, demonstrating a comparable rise in fluorescence intensity within the specified CT range, indicating successful amplification and detection of the OXA-51 gene. Both amplification plots reflect the specificity and efficiency of the RT-PCR assay, ensuring accurate identification of carbapenemase genes critical to understanding resistance mechanisms in *Enterobacteriaceae* isolates.

## Discussion

In clinical settings, *Enterobacteriaceae* isolates are the most prevalent encountered organisms. The line of medications that is kept in reserve is the carbapenem group. The organisms exhibit heightened resistance to carbapenems, hence restricting the clinician’s options for treatment. Therefore, it is critical to understand the prevalence and mechanism of carbapenem resistance in *Enterobacteriaceae*. The current investigation was conducted with the previously mentioned view in mind. In this study, the genotypic approach was used to identify the prevalence of CRE. Carbapenem-resistant *Escherichia coli* is more prevalent because of the irrational use of antibiotic drugs. The urinary tract is most frequently infected by *Escherichia* coli because the uropathogenic *Escherichia coli* have specific adhesion factors like fimbrial antigen, which causes adhesion and colonisation of the urinary tract [12].

By administering the right narrow-spectrum antibiotic for the right infection, this can be avoided. These investigations revealed a concerning trend of CRE, which should be avoided by using antibiotics appropriately and identifying resistant strains. Our institution’s low CRE prevalence may be attributable to the way infection control procedures are implemented [13]. Among the 22 CRE isolates, which were phenotypically confirmed by VITEK-2, (13, 59%) were isolated from males, followed by females (nine, 41%). This exactly correlates with the study done by Pawar et al. [14], where the highest prevalence was seen in males (65.2%) followed by females (30.76%) respectively. The highest prevalence of CRE was seen in 45-65 years (45%), which was similar to the study above [14], which said that 41-60 years (37.05%) showed high CRE prevalence.

The antibiotic-resistant pattern among the 22 CRE isolates from our study showed similar findings to a study by Sharma et al. [15], wherein *Escherichia coli* (67.9%) showed a higher resistance rate than *Klebsiella* spp. (66.9%). According to AlTamimi et al. [16], compared to *Escherichia coli *(19.2%), *Klebsiella pneumonia*e (70.5%) had a higher resistance pattern. This results from mutated porin channels, elevated efflux pump and the synthesis of carbapenemase.

In another study done by Datta et al. [17], the most common CRE isolates were *Klebsiella oxytoca* (46.15%), which is followed by *Escherichia coli* (26.92%). Similarly, contrary to this, Trojan et al. [18] said that *Escherichia coli* was the most common pathogen isolated (51.2%), followed by *Klebsiella pneumoniae* (11.6%). Molecular characterisation of these 22 isolates by Multiplex RT-PCR showed NDM (63.63%) was the most common gene detected from isolates. The other genes detected were OXA-23 (59.09%), VIM (40.9%), KPC (13.63%), OXA-51 (18.18%) and OXA-58 (13.63%), as shown in Figures 2-3. This correlated with the studies conducted by Mohan et al. [19], where the NDM-1 gene was present in nearly 47 (53.4%) isolates, VIM in 19 (24.4%), IMP in one (1.1%), and none had KPC. A similar study was conducted by Delarampour et al. [20], where 75% of CRE produced NDM. In another study conducted by Nagaraj et al. [2], NDM was detected in the majority of carbapenem-resistant *Klebsiella pneumoniae* (75%) and *Escherichia coli *(66.6%). In another study conducted by Djahmi et al. [21], the OXA-48 group was the most common carbapenemase detected. In another study conducted by Shin et al. [22], OXA-23 was the most common carbapenemase detected.

Among the 22 carbapenemase-producers, seven (31%) isolates were exclusive MBL producers, five (22%) isolates were exclusively class D carbapenemase producers and 10 (45%) isolates were co-producers of MBL and classes A and D carbapenemases. This is in concordance with the study conducted by Anandan et al. [11], where co-production of both OXA-48 and NDM was seen in 15 (12.5%) isolates.

Among the nine *Escherichia coli*, NDM (88.8%) was the major carbapenemase gene detected, followed by VIM (44.4%). Among nine *Klebsiella* spp., the major gene detected was NDM (66.5%), followed by VIM (62.5%). This was consistent with research by Veeraraghavan et al. [23], which found that *Escherichia coli* accounted for 75% of NDM cases, with *Klebsiella* spp. accounting for 67% of cases. VIM and NDM are more commonly isolated clinically relevant MBL. The more prevalent gene that is epidemiologically linked to India is NDM, and the more common carbapenemase gene globally soon will also be NDM [19]. PCR offers 100% sensitivity and specificity as well; however, it is more expensive and not affordable for everyone. With all of the aforementioned findings, 8.8% of the *Enterobacteriaceae* in our investigation were carbapenem-resistant. In our tertiary care settings, carbapenemase synthesis was reported to be the most common cause of the resistance mechanism. The presence of the genes NDM and VIM revealed that MBL predominated among these carbapenemases. 

Polymyxins like colistin and polymyxin b, along with tigecycline, are extensively used to treat infections caused by carbapenemase-producing strains. Fosfomycin can be effectively used to treat lower urosepsis caused by *Escherichia coli*. However, molecular characterisation of carbapenem-resistant isolates can guide to several colistin and tigecycline-sparing options as these antibiotics cannot be used in patients with altered renal parameters, which are very commonly associated with sepsis. Ceftazidime-avibactam is a combination of a newly developed β-lactamase inhibitor called avibactam and a well-known broad-spectrum cephalosporin called ceftazidime. It works against β-lactamases of classes A, C and some D [15,23]. Avibactam is active against KPC-type carbapenemases and OXA enzymes.

The treatment options include the use of polymyxins, fosfomycin, and aminoglycosides, which are older antibiotics that have been used rarely because of toxicity and/or efficacy concerns. The additional treatment strategies include combination therapy or optimisation of dosing regimens. The following antibiotics such as colistin, aminoglycosides, tigecycline, fosfomycin, ceftazidime/avibactam and ceftolozane/tazobactam are often used to treat quadruple MRGN pathogens. Imipenem-relebactam and meropenem-vaborbactam are used to restore the activity of β-lactams against β-lactamase-producing Gram-negative bacteria, particularly to inactivate KPCs and other carbapenemase-producing *Enterobacterales* and also effective against *Pseudomonas aeruginosa* isolates [24]. The most commonly used in vitro active and potentially useful drugs are newer inhibitor combinations, ceftazidime-avibactam, colistin, fosfomycin, gentamicin, amikacin and tigecycline. Plazomycin, eravacycline and cefiderocol are the newer or emerging options for the treatment.

The study has notable strengths and limitations. Among its strengths, the use of RT-PCR for the phenotypic and genotypic detection of specific carbapenemase genes ensures robust and accurate results, providing valuable regional insights into carbapenem resistance patterns. However, some limitations that warrant attention include the lack of investigation into other resistance mechanisms, such as efflux pumps or porin channel loss, which may also contribute to carbapenem resistance. The cross-sectional design limits the ability to assess temporal changes in resistance patterns or evaluate the impact of interventions over time. Furthermore, as the study was conducted in a single tertiary care hospital, its findings may not be generalisable to regional or national levels. These factors should be considered when interpreting the results and designing future research.

## Conclusions

This study determined the prevalence of carbapenem-resistant Enterobacteriaceae (CRE) to be 8.8% and identified genetic mechanisms underlying their resistance. CRE infections, originating from diverse sources, are often associated with prolonged hospital stays due to underlying comorbid conditions. The findings underscore the critical role of laboratories in identifying resistance mechanisms and incidence to inform infection control strategies and antibiotic stewardship efforts. With treatment options limited to agents such as Polymyxins, Fosfomycin, and Aminoglycosides, the study highlights the urgent need for rational antibiotic use, stringent infection control measures, and adherence to antimicrobial stewardship policies. Moreover, the routine monitoring of carbapenemase-encoding genes is vital to track and mitigate resistance. 
